# Supplementary material for: Single-cell normalization and association testing unifying CRISPR screen and gene co-expression analyses with Normalisr
Source: Nat Commun. 2021 Nov 4;12:6395. doi: 10.1038/s41467-021-26682-1 (PMC8568964; doi:10.1038/s41467-021-26682-1)
Supplement: Supplementary file 1 — Supplementary Information [file 41467_2021_26682_MOESM1_ESM.pdf]

Supplementary Information for Single-cell normalization and association testing unifying CRISPR screen and gene co-expression analyses with Normalisr

Lingfei Wang✉

Broad Institute of MIT and Harvard, Cambridge, USA

Center for Computational and Integrative Biology, Massachusetts General Hospital, Boston, USA

Molecular Pathology Unit and Center for Cancer Research, Massachusetts General Hospital Research Institute, Charlestown, USA

1 Concepts

1.1 Unbiased cellular summary covariates

Given the read counts of genes $i = 1, \ldots, n_g$ for a cell as vector $\mathbf{g} = \{g_i\}$ and no additional information to favor one gene over another (*a.k.a* unbiased in the biological definition instead of its statistical definition), the L^0 norm of vector $\mathbf{g}$ is $\#_i g_i \neq 0$. Its L^1 norm is $\sum_i g_i$. They each equate the number of zero-read genes and the log total read count after a linear and a log transformation. The use of log total read count instead of total read count as a covariate is natural because the variables themselves (log expression) are in log space.

2 Derivations

2.1 Minimum mean square error (MMSE) estimator for $\ln p$ in $Binom(n, p)$

See https://en.wikipedia.org/wiki/Beta_distribution#Geometric_mean.

Consider a single data point k sampled from the binomial distribution $Binom(n, p)$, in which n is known. Without any other information, we assume $p \sim U(0, 1) = Beta(1, 1)$ follows a standard uniform prior distribution. Then the posterior distribution for p is $Beta(k + 1, n - k + 1)$ as the conjugate of binomial distribution. The posterior probabilistic density function is

$$P(p) = \frac{p^{\alpha-1}(1-p)^{\beta-1}}{B(\alpha, \beta)}, \tag{1}$$

where $\alpha = k + 1$, $\beta = n - k + 1$, $B(\alpha, \beta) = \Gamma(\alpha)\Gamma(\beta)/\Gamma(\alpha + \beta)$, and Γ is the Gamma function.

✉E-mail: lwang55@mgh.harvard.edu

The MMSE estimator for $\ln p$ is

$$\begin{aligned}
\widehat{\ln p} &\equiv \mathbb{E}\ln p \\
&= \frac{1}{B(\alpha,\beta)}\int_0^1 x^{\alpha-1}(1-x)^{\beta-1}\ln x dx \\
&= \frac{1}{B(\alpha,\beta)}\frac{\partial}{\partial\alpha}\int_0^1 x^{\alpha-1}(1-x)^{\beta-1}dx \\
&= \frac{1}{B(\alpha,\beta)}\frac{\partial B(\alpha,\beta)}{\partial\alpha} \\
&= \frac{\partial \ln B(\alpha,\beta)}{\partial\alpha} \\
&= \frac{\partial \ln\Gamma(\alpha)}{\partial\alpha} - \frac{\partial \ln\Gamma(\alpha+\beta)}{\partial\alpha} \\
&= \psi(\alpha) - \psi(\alpha+\beta), \qquad (2)
\end{aligned}$$

where ψ is the digamma function.

3 Proofs

3.1 Inexistence of unbiased estimator for $\ln p$ in $Binom(n,p)$

Consider the random variable $X \sim Binom(n,p)$ with fixed n and p, where $n \in \mathbb{N}^*$ is known and $p \in (0,1)$ is unknown. To find an unbiased estimator for $\ln p$, assume it exists as $\widehat{\ln p}(n;X)$. Since the domain of the unbiased estimator is $\{0,1,\ldots,n\}$, we can rewrite it as

$$\widehat{\ln p}(n;X) = \sum_{x=0}^{n} \delta_{X,x} v_x(n), \qquad \text{where } v_x(n) \equiv \widehat{\ln p}(n;x), \qquad (3)$$

and $\delta_{i,j} = 1$ if $i = j$ and 0 otherwise is the Kronecker delta function. This represents $\widehat{\ln p}(n;X)$ as a value vector $\mathbf{v}(n) \equiv (v_0(n),\ldots,v_n(n))$ of size $n+1$. Solving $\mathbf{v}(n)$ would solve the unbiased estimator.

The definition of unbiased estimator requires that for all $p \in (0,1)$,

$$\begin{aligned}
\ln p &= \mathbb{E}_X \widehat{\ln p}(n;X) \\
&= \sum_{x=0}^{n} P(X = x \mid n,p)\,\widehat{\ln p}(n;x) \\
&= \sum_{x=0}^{n} \frac{n!p^x(1-p)^{n-x}}{x!(n-x)!}\widehat{\ln p}(n;x) \\
&= \sum_{x=0}^{n} \frac{n!p^x(1-p)^{n-x}}{x!(n-x)!} v_x(n). \qquad (4)
\end{aligned}$$

Note the *r.h.s* is a polynomial of p up to order n, whose coefficients are linear functions of $\mathbf{v}(n)$. However, the Taylor expansion of the *l.h.s* is an infinite order polynomial with nonvanishing coefficients. Therefore a solution for $\mathbf{v}(n)$ that satisfies this equation for all $p \in (0, 1)$ does not exist. Correspondingly the unbiased estimator $\widehat{\ln p}(n; X)$ for $\ln p$ does not exist.

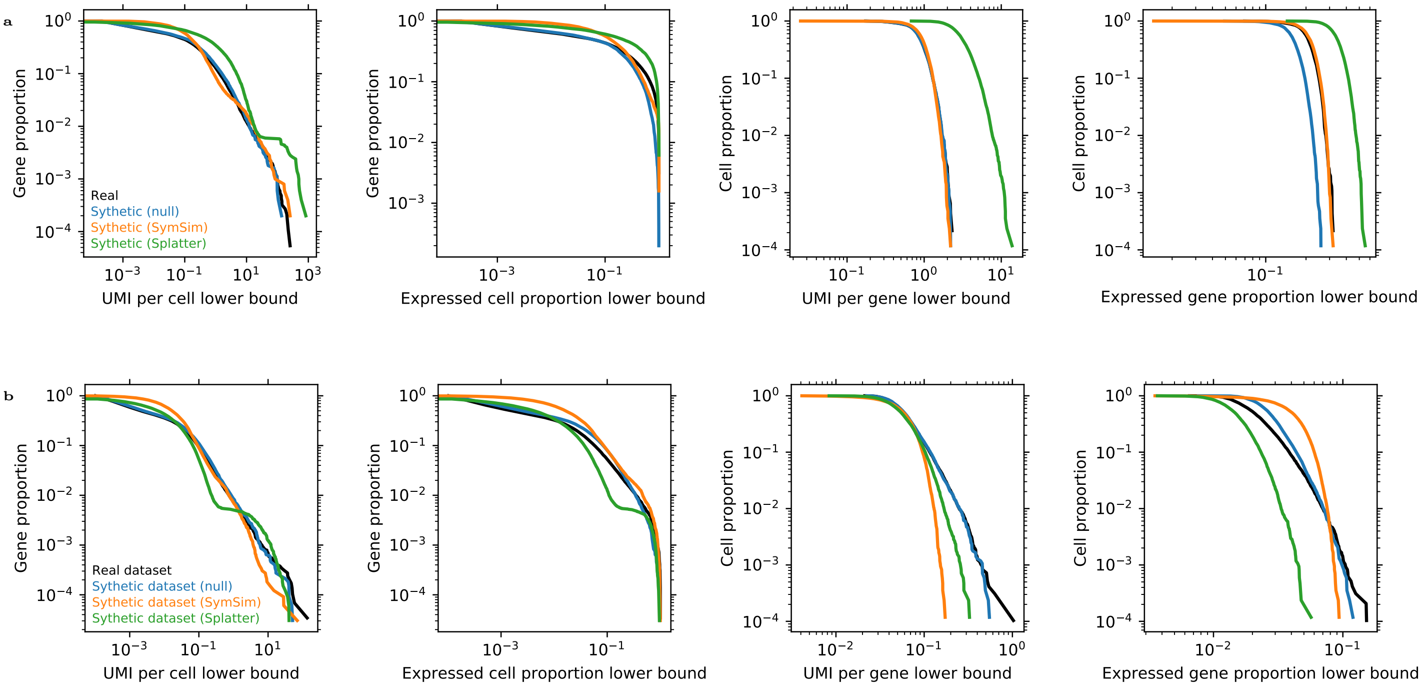

Supplementary Figure 1: **Synthetic datasets from different simulation methods mimicked the read count distributions of the real dataset with different similarities in survival functions.** **a** For Perturb-seq, 5,017 genes in 8,472 cells were simulated from 18,583 genes in 4,622 cells in the real dataset. **b** For MARS-seq, 32,854 genes in 8,472 cells were simulated from 29,161 genes in 9,760 cells in the real dataset. Expressed cell for a given gene (x axis in columns 2 and 4) is defined as those cells with non-zero read count for the gene. Colors indicate different simulation methods.

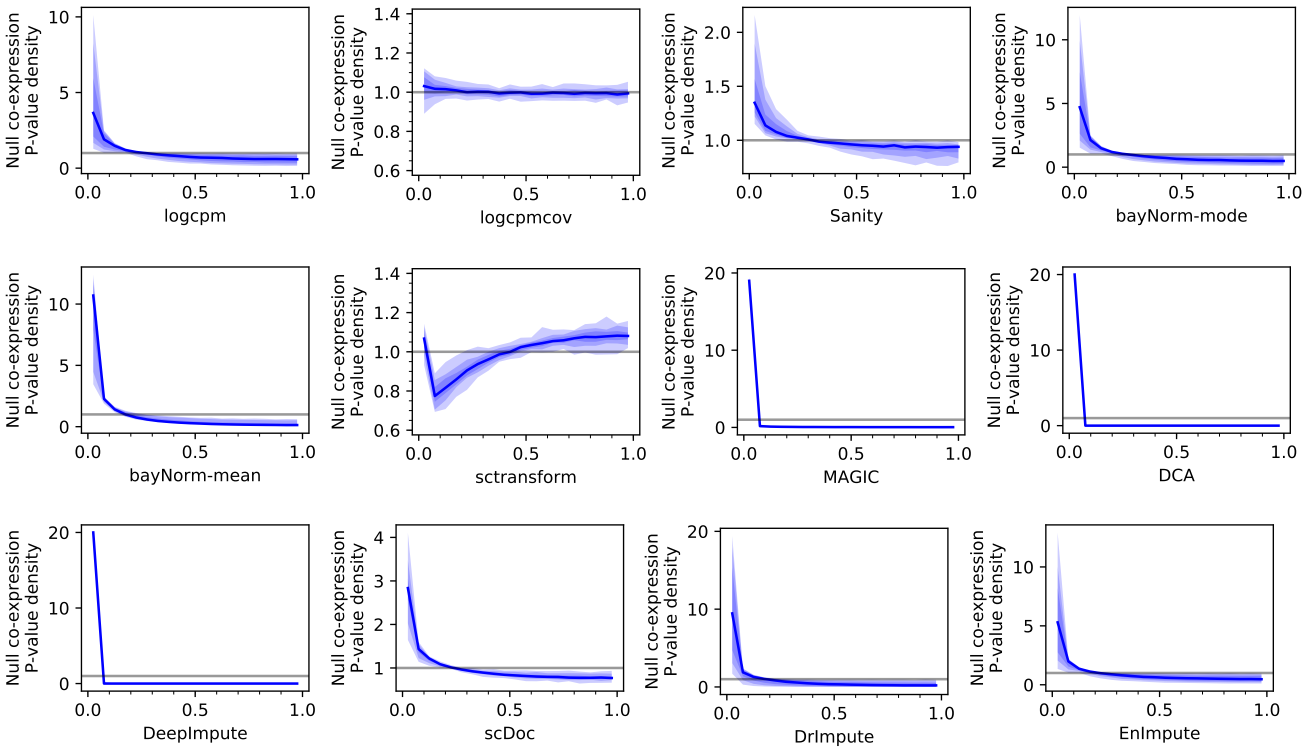

Supplementary Figure 2: **Other methods failed to model the sparsity in the multinomial mRNA sampling process and recovered distorted null P-values of single-cell co-expression (X) as shown by histogram density (Y)**. Left to right and top to bottom: log(CPM+1), log(CPM+1) with cellular summary covariates, Sanity, bayNorm-mode, bayNorm-mean, sctransform, MAGIC, DCA, DeepImpute, scDoc, DrImpute, and EnImpute. Genes were split into 10 equal bins from low to high expression. The null P-value distribution of co-expression between each bin pair formed a separate histogram curve. Central curve shows the median of all histogram curves. Shades show 50%, 80%, and 100% of all histograms. Gray line indicates uniform distribution. LogCPM's 0-biased P-values indicated that the null synthetic dataset could recapitulate the technical confounding effect from the multinomial sampling process of RNA sequencing. See Fig. 2d for the panel for Normalisr and the description of drawing style.

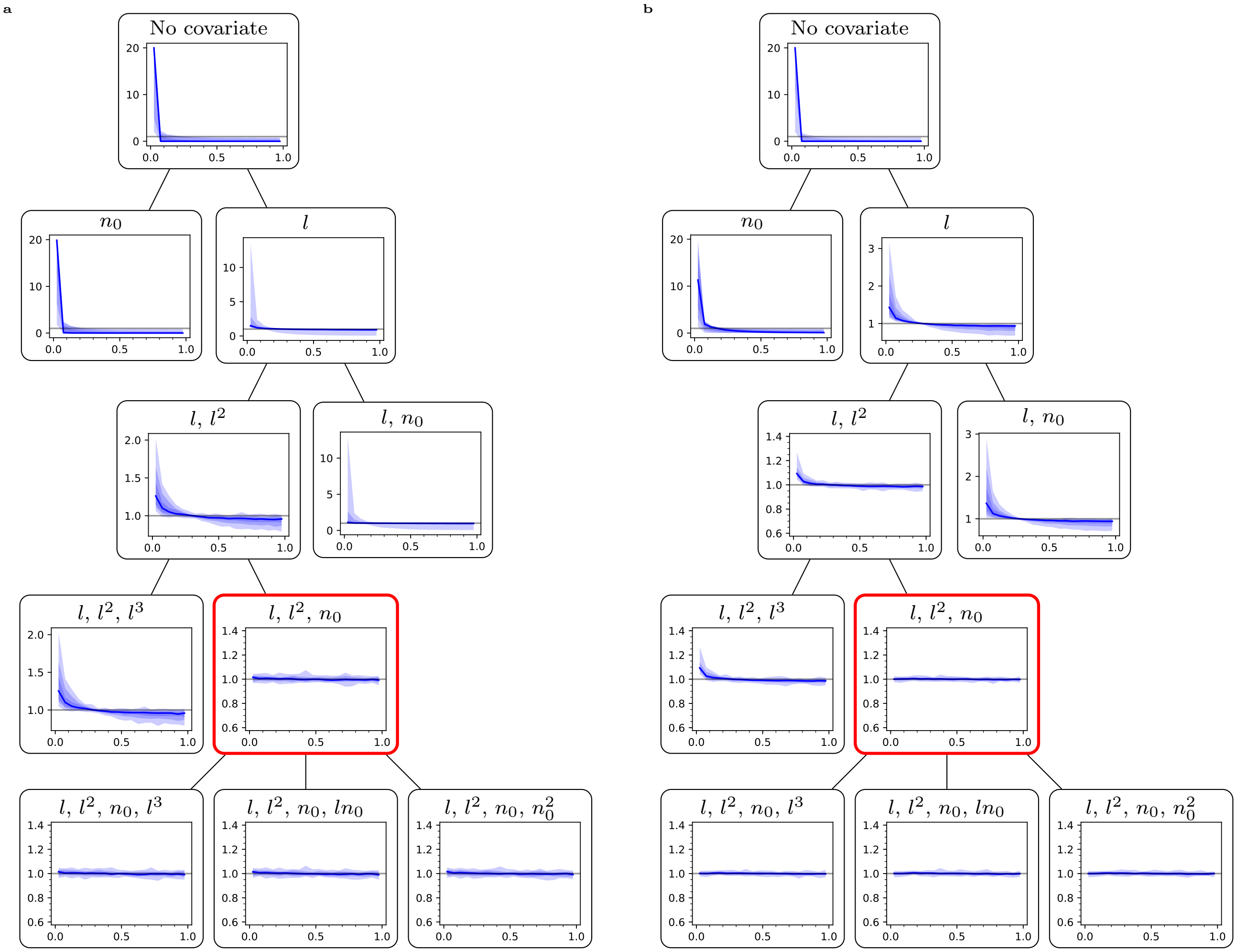

Supplementary Figure 3: **Decision tree to introduce covariates on the Perturb-seq dataset (a) or to validate them on the MARS-seq dataset (b) iteratively as a Taylor expansion.** Each step (top to bottom) shows the covariates (l: total log read count in each cell, n_0: number of 0-read genes in each cell, besides constant intercept) and the resulting histograms (Y) of null co-expression P-values (X), based on the same drawing style as in Fig. 2c. The covariate set was optimized towards a uniform distribution of null co-expression P-values from the synthetic null dataset. Red: the covariate set with the best P-value histogram and fewest covariates was selected for Normalisr from the Perturb-seq dataset and confirmed in the MARS-seq dataset.

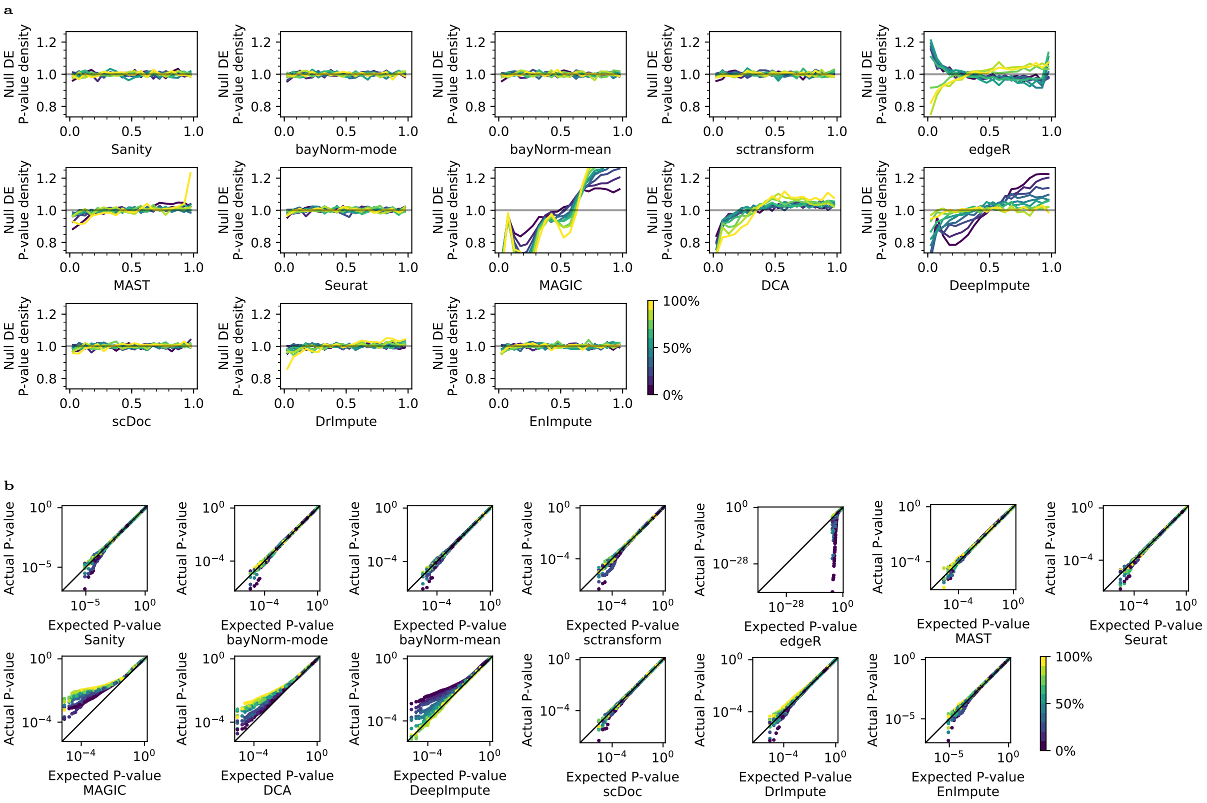

Supplementary Figure 4: **Method performances vary in recovering uniformly distributed null P-values in single-cell DE. a** Distribution of null P-values in single-cell DE (X) as shown by histogram density (Y). **b** Quantile-quantile plot of expected (X) and actual (Y) null P-values. Each dot represents the FPR (Y) at the corresponding significance cutoff for P-value (X). Genes were evaluated in 10 bins from low to high expression (color). The panels for Normalisr are in Fig. 2a.

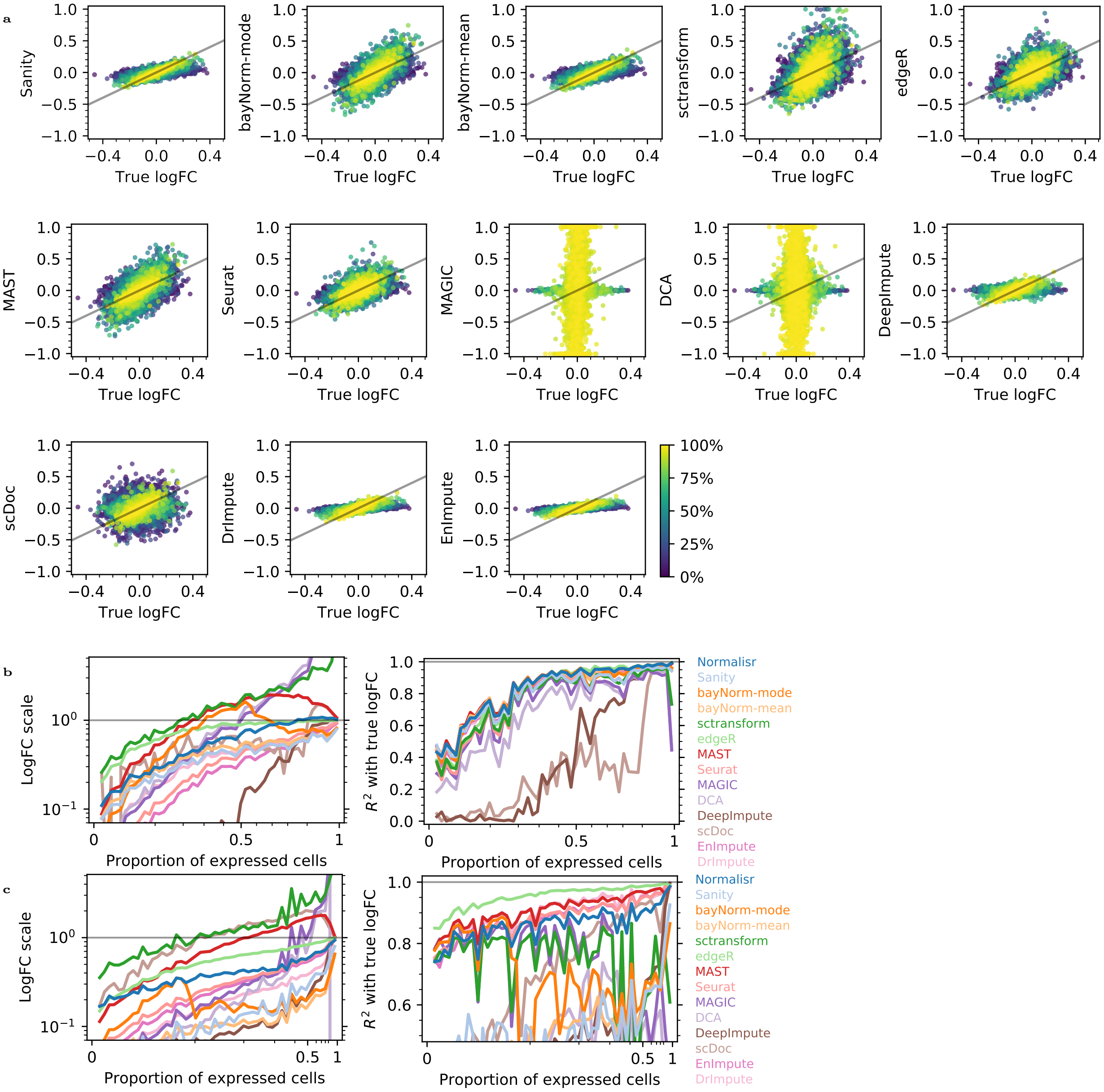

Supplementary Figure 5: **Normalisr had low bias and variance in logFC estimation on different synthetic datasets based on the Perturb-seq dataset**. **a** Recovered logFCs (Y) were compared against the synthetic ground-truth (X) for genes (dot) from low to high expression (color) for different methods on the synthetic null dataset. Gray lines indicate X=Y. The panel for Normalisr is in Fig. 3a. **bc** Normalisr accurately recovered logFCs with low bias (left, Y as regression coefficient) and low variance (right, Y as R^2) when evaluated on synthetic datasets from Splatter (**b**) and SymSim (**c**). Horizontal gray lines indicate bias- or variance-free performance.

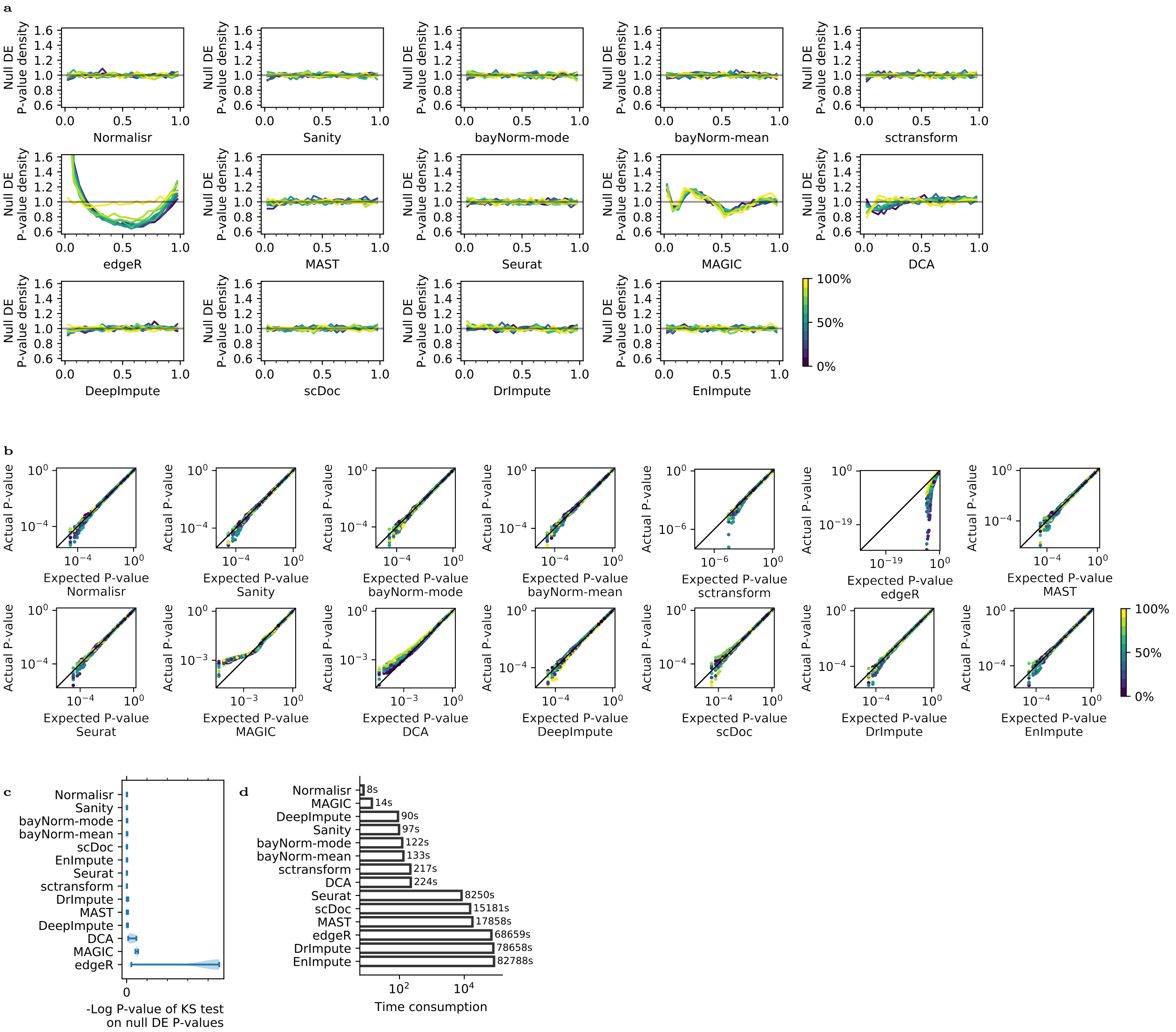

Supplementary Figure 6: **Null DE evaluation results were reproducible on the MARS-seq dataset of dysfunctional T cells from frozen human melanoma tissue**. Evaluations were reproduced for Fig. 2a and Fig. S4 (**ab**), Fig. 2b (**c**), and Fig. 2c (**d**). **abc** Normalisrhad uniformly distributed null P-values in single-cell DE. **d** Normalisr was much faster than other methods.

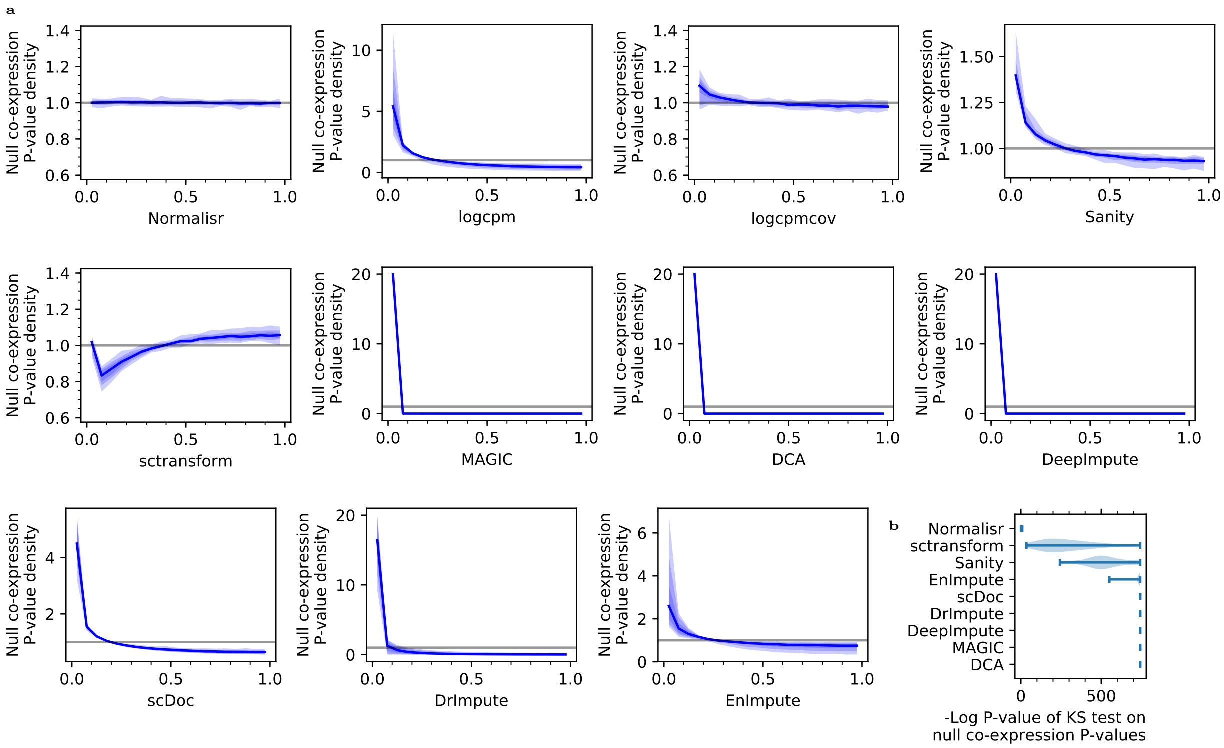

Supplementary Figure 7: **Null co-expression evaluation results were reproducible on the MARS-seq dataset of dysfunctional T cells from frozen human melanoma tissue**. Evaluations were reproduced for Fig. 2d and Fig. S2 (**a**), and Fig. 2e (**b**). Only Normalisr had uniformly distributed null P-values in single-cell co-expression from the synthetic data that mimicked MARS-seq of dysfunctional T cells from frozen human melanoma tissues. BayNorm failed parts of the evaluations with undocumented errors and could not be compared.

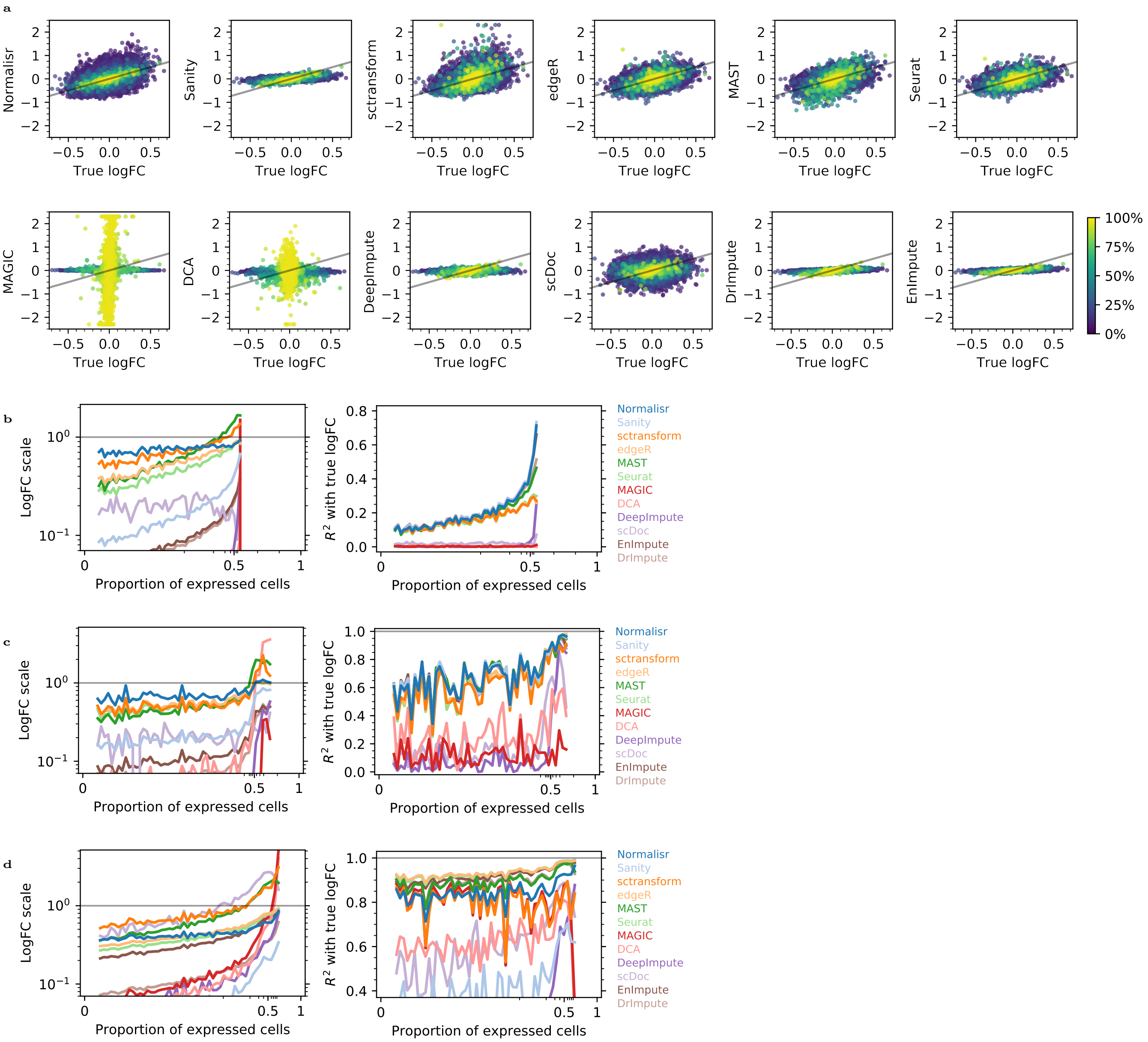

Supplementary Figure 8: **DE logFC evaluation results were reproducible on the MARS-seq dataset of dysfunctional T cells from frozen human melanoma tissue**. Evaluations were reproduced for Fig. 3a and Fig. S5a (**a**), Fig. 3b (**b**), and Fig. S5bc (**cd**). **a** Normalisr accurately recovered logFCs (Y) when compared against the ground-truth (X) for genes from low to high expression (color) on the synthetic null dataset. Gray line indicates X=Y. **bcd** Normalisr accurately recovered logFCs with low bias (left, Y as regression coefficient) and low variance (right, Y as R^2) when evaluated on synthetic datasets from null co-expression (**b**), Splatter (**c**), and SymSim (**d**). Horizontal gray lines indicate bias- or variance-free performance. BayNorm failed parts of the evaluations with undocumented errors and could not be compared.

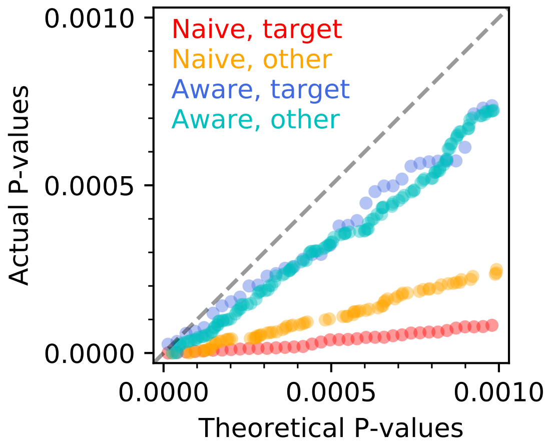

Supplementary Figure 9: **Quantile-quantile plot of theoretical (X) and actual (Y) P-values of CRISPRi DE (Fig. 1c) from NTC gRNAs**. DE was performed separately with competition-**naive** and -**aware** methods and separately for genes **target**ed by positive control gRNAs at the TSS and **other** genes. Data points were randomly down-sampled to avoid overly dense plots.

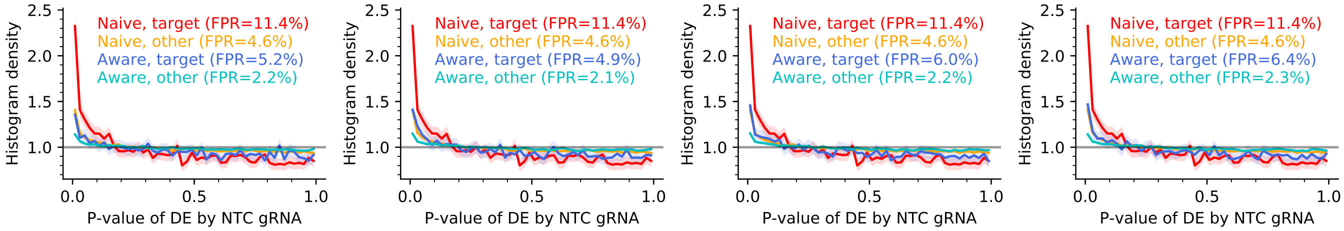

Supplementary Figure 10: **Top principal components of gRNA variation could account for most of the confounding effects on the small-scale CRISPRi screen**. Density histograms (Y) of P-values (X) from the competition-aware method remained almost identical between considering (left-to-right) all other gRNAs and their top 500, 200, or 100 principal components. Gray lines indicate the expected uniform distribution for null P-value. Shades indicate absolute errors estimated as $2\sqrt{N+1}$, where N is the count in each bin.

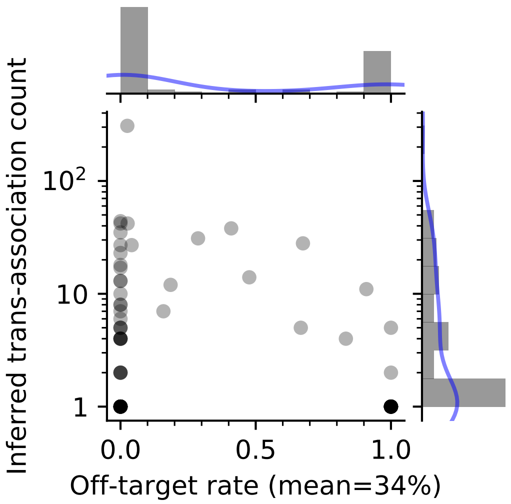

Supplementary Figure 11: **CRISPRi off-target effects were weaker than genuine trans-associations**. Estimated mean off-target rate (X) reduced to 34% at $Q \leq 10^{-5}$ for significant trans-associations.

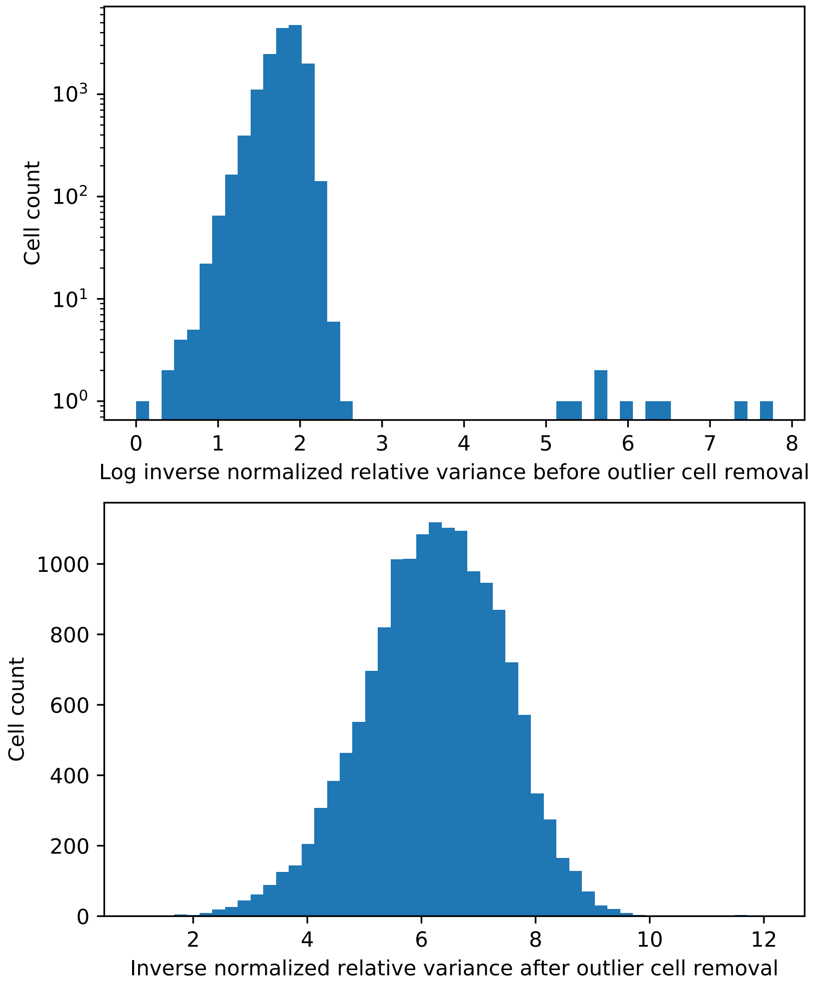

Supplementary Figure 12: **MARS-seq melanoma dataset contained few outlier cells/samples with very low variances**. The histograms (Y) show the distributions of inverse normalized relative variances (X) for each cell in the dysfunctional *v.s.* naive T cell setting. Outlier cells had distinctively lower variances than the major population (top). Our outlier removal strategy successfully removed these outliers (bottom).

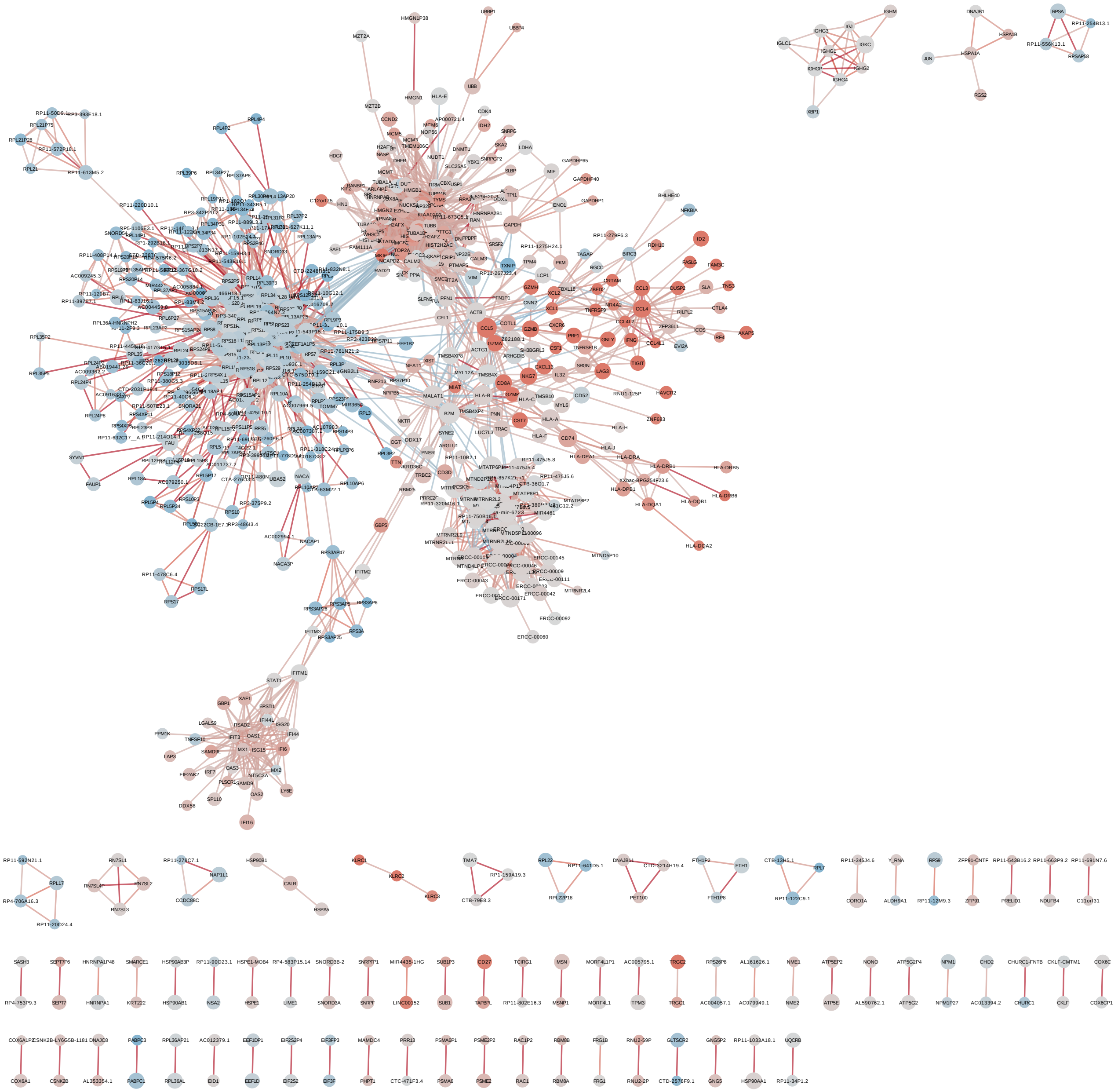

Supplementary Figure 13: **Single-cell transcriptome-wide co-expression network of dysfunctional T cells from human melanoma before GO program removal**. Legend is in Fig. S14. Zooming in on a digital device is advised.

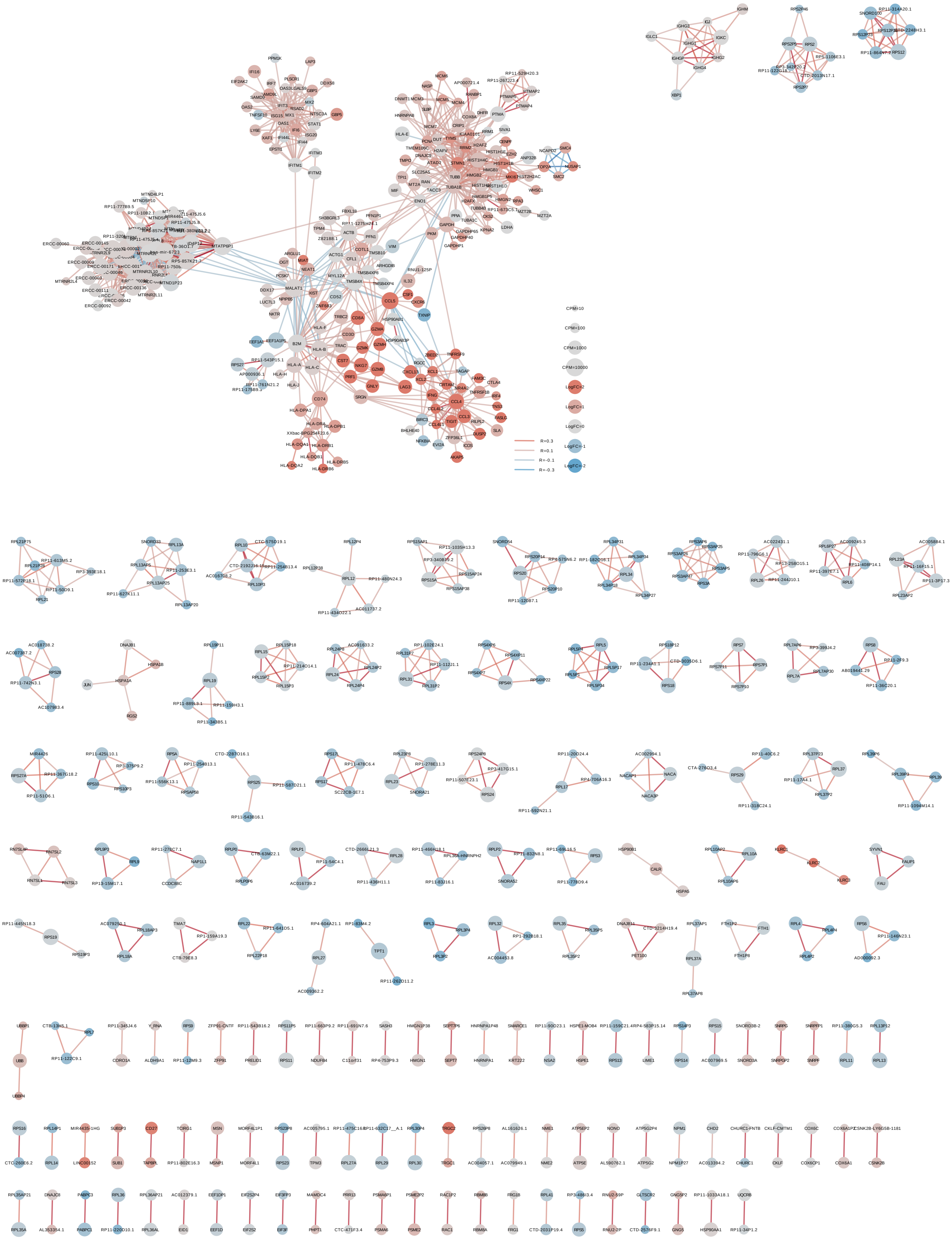

Supplementary Figure 14: **Single-cell transcriptome-wide co-expression network of dysfunctional T cells from human melanoma after GO program removal**. Removed GO programs were cytosolic part (GO:0044445) and chromosome condensation (GO:0030261). Fig. 7c further removed the non-coding cluster and the minor connected components. Zooming in on a digital device is advised.

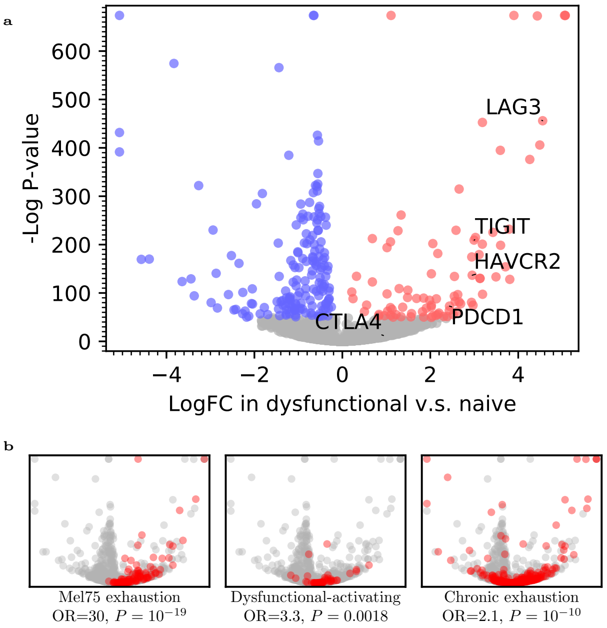

Supplementary Figure 15: **Normalisr identified DEs between dysfunctional and naive T cells in human melanoma MARS-seq**. **a** Normalisr detected expression changes associated with T cell dysfunction, shown in a volcano plot of single-cell DE between logFC (X) and -log P-value (Y, Fig. 1c). Red/blue indicates up-/down-regulated genes (Benjamini-Hochberg Q-value $\leq 10^{-20}$). **b** Up-regulated genes significantly overlapped with published T cell dysfunction signature gene sets (red) in terms of odds ratio (OR) and two-sided hypergeometric P-value, including (left-to-right) the *Mel75* exhaustion signature in scRNA-seq of human melanoma [49], the dysfunctional-activating signature in bulk RNA-seq of implanted mouse MC38 tumors [47], and the chronic T cell exhaustion signature in bulk RNA-seq of lymphocytic choriomeningitis virus infected mice [48].
