# Supplementary material for: Single-cell normalization and association testing unifying CRISPR screen and gene co-expression analyses with Normalisr
Source: Nat Commun. 2021 Nov 4;12:6395. doi: 10.1038/s41467-021-26682-1 (PMC8568964; doi:10.1038/s41467-021-26682-1)
Supplement: Supplementary file 3 — Description of Additional Supplementary Files [file 41467_2021_26682_MOESM3_ESM.pdf]

**Description of Additional Supplementary Files**

File Name: Supplementary Data 1
Description: Gene, cell, and gRNA counts of all studies.

File Name: Supplementary Data 2
Description: Unexpected associations and potential off-target effects of NTC gRNAs in CROP-seq screen.

File Name: Supplementary Data 3
Description: Gene regulatory network inferred from CROP-seq screen.

File Name: Supplementary Data 4
Description: Top GO enrichments of inferred targets of top up-/down-regulation favored regulators in CROP-seq screen.

File Name: Supplementary Data 5
Description: Significant gene ontology enrichments of top 100 principal genes in the co-expression network of dysfunctional T cells, for different numbers of GO covariates in iterative GO pathway removal.

File Name: Supplementary Data 6
Description: Differential gene expression result in dysfunctional v.s. naive T cells.

File Name: Supplementary Data 7
Description: Node and edge properties of Fig. 7c, Fig. S13, and Fig. S14.

File Name: Supplementary Data 8
Description: Top gene ontology enrichments of the cell cycle and type I interferon response clusters in the co-expression network of dysfunctional T cells.

File Name: Supplementary Data 9
Description: Over-abundance of co-expression edges between genes in the same GO or KEGG pathways.
