# Supplementary material for: Single-cell normalization and association testing unifying CRISPR screen and gene co-expression analyses with Normalisr
Source: Nat Commun. 2021 Nov 4;12:6395. doi: 10.1038/s41467-021-26682-1 (PMC8568964; doi:10.1038/s41467-021-26682-1)
Supplement: Supplementary file 5 — Reporting Summary [file 41467_2021_26682_MOESM5_ESM.pdf]

Corresponding author(s): Lingfei Wang

Last updated by author(s): Oct 7, 2021

Reporting Summary

Nature Research wishes to improve the reproducibility of the work that we publish. This form provides structure for consistency and transparency in reporting. For further information on Nature Research policies, see our Editorial Policies and the Editorial Policy Checklist.

Statistics

For all statistical analyses, confirm that the following items are present in the figure legend, table legend, main text, or Methods section.

n/a	Confirmed	
☐	☒	The exact sample size (*n*) for each experimental group/condition, given as a discrete number and unit of measurement
☐	☒	A statement on whether measurements were taken from distinct samples or whether the same sample was measured repeatedly
☐	☒	The statistical test(s) used AND whether they are one- or two-sided<br>*Only common tests should be described solely by name; describe more complex techniques in the Methods section.*
☐	☒	A description of all covariates tested
☐	☒	A description of any assumptions or corrections, such as tests of normality and adjustment for multiple comparisons
☐	☒	A full description of the statistical parameters including central tendency (e.g. means) or other basic estimates (e.g. regression coefficient) AND variation (e.g. standard deviation) or associated estimates of uncertainty (e.g. confidence intervals)
☐	☒	For null hypothesis testing, the test statistic (e.g. *F*, *t*, *r*) with confidence intervals, effect sizes, degrees of freedom and *P* value noted<br>*Give P values as exact values whenever suitable.*
☐	☒	For Bayesian analysis, information on the choice of priors and Markov chain Monte Carlo settings
☒	☐	For hierarchical and complex designs, identification of the appropriate level for tests and full reporting of outcomes
☐	☒	Estimates of effect sizes (e.g. Cohen's *d*, Pearson's *r*), indicating how they were calculated

Our web collection on statistics for biologists contains articles on many of the points above.

Software and code

Policy information about availability of computer code

Data collection	This study uses published datasets and does not involve data collection.
Data analysis	sctransform 0.2.1, bayNorm 1.2.0, Sanity 1.0, edgeR 3.26.8, MAST 1.10.0, DeepImpute 1.2, scDoc 0.0.0.9, DrImpute 1.0, EnImpute 1.1, Seurat 4.0.3, SymSim 0.0.0.9, Splatter 1.14.1, MAGIC 1.2.1, DCA 0.2.3, goatools 0.8.4, mygene 3.1.0, bioservices 1.7, fdrtool 1.2.15. The custom software 'Normalisr' is reported in 'Code and Software Submission Checklist' and available at https://github.com/lingfeiwang/normalisr.

For manuscripts utilizing custom algorithms or software that are central to the research but not yet described in published literature, software must be made available to editors and reviewers. We strongly encourage code deposition in a community repository (e.g. GitHub). See the Nature Research guidelines for submitting code & software for further information.

Data

Policy information about availability of data

All manuscripts must include a data availability statement. This statement should provide the following information, where applicable:
- Accession codes, unique identifiers, or web links for publicly available datasets
- A list of figures that have associated raw data
- A description of any restrictions on data availability

We downloaded published scRNA-seq datasets from Gene Expression Omnibus with accession numbers: GSM2406681 (Perturb-seq), GSE123139 (MARS-seq), GSE120861 (CROP-seq), GSM2138664 (shRNA DDX3X knock-down, ENCODE identifier: ENCSR000KYM), and GSM2138876 (shRNA control, ENCODE identifier: ENCSR913CAE). SCEPTRE results were downloaded from https://drive.google.com/drive/folders/1ynZRMvGtFxfBiD0zAcuIYjNeS8Jj4AP9 in Ref [13].

Field-specific reporting

Please select the one below that is the best fit for your research. If you are not sure, read the appropriate sections before making your selection.

[x] Life sciences [ ] Behavioural & social sciences [ ] Ecological, evolutionary & environmental sciences

For a reference copy of the document with all sections, see nature.com/documents/nr-reporting-summary-flat.pdf

Life sciences study design

All studies must disclose on these points even when the disclosure is negative.

Sample size	This study uses published datasets and does not involve sample collection. Simulated dataset sizes were decided with random key strokes and with replication in different random sizes that cover a wide range of realistic scenarios and therefore sufficient for the analysis.
Data exclusions	We removed low-read genes (expressed in <500 cells or <2% of cells for MARS-seq and expressed in <50 cells or <2% of cells for others) due to insufficient information for statistical inference. We removed low-read cells (with <500 UMIs or 100 expressed genes) due to low confidence in quality. We removed low-variance outlier cells (two-sided P-value <1E-10) for MARS-seq due to low confidence in quality. Gene and cell exclusion rationales were pre-established. Details of data exclusion were documented in Methods.
Replication	Null DE were replicated 100 times across random groupings. Analysis from SymSim or Splatter simulations were performed 4 times. The full results were in agreement and shown together. Gene regulatory networks reconstructed from two single-cell CRISPR experiments were in agreement (Figure 6a). Method performance evaluation analysis in sensitivity, specificity, differential expression, and co-expression was confirmed with overall agreement on the MARS-seq dataset (Supplementary Figures 1,3,6,7,8) and separately not shown for GSE106510. Replication with different sample counts were described in main text.
Randomization	Batch effects were accounted for as covariates. This includes sequencing, amplification, and sampling batches. This study uses published datasets and randomization cannot be modified.
Blinding	Blinding is not applicable in this study because group allocations were pre-determined by published datasets.

Reporting for specific materials, systems and methods

We require information from authors about some types of materials, experimental systems and methods used in many studies. Here, indicate whether each material, system or method listed is relevant to your study. If you are not sure if a list item applies to your research, read the appropriate section before selecting a response.

Materials & experimental systems

n/a	Involved in the study	
[x]	[ ]	Antibodies
[x]	[ ]	Eukaryotic cell lines
[x]	[ ]	Palaeontology and archaeology
[x]	[ ]	Animals and other organisms
[x]	[ ]	Human research participants
[x]	[ ]	Clinical data
[x]	[ ]	Dual use research of concern

Methods

n/a	Involved in the study	
[x]	[ ]	ChIP-seq
[x]	[ ]	Flow cytometry
[x]	[ ]	MRI-based neuroimaging
